# The energetic cost of parasitism in a wild population

**DOI:** 10.1098/rspb.2018.0489

**Published:** 2018-05-30

**Authors:** Olivia Hicks, Sarah J. Burthe, Francis Daunt, Mark Newell, Adam Butler, Motohiro Ito, Katsufumi Sato, Jonathan A. Green

**Affiliations:** 1 School of Environmental Sciences, University of Liverpool, Liverpool L69 3GP, UK; 2 Centre for Ecology and Hydrology, Bush Estate, Penicuik, Midlothian, EH26 0QB, UK; 3 Biomathematics and Statistics Scotland, James Clerk Maxwell Building, The King's Buildings, Peter Guthrie Tait Road, Edinburgh EH9 3FD, UK; 4 Department of Applied Biosciences, Faculty of Life Sciences, Tokyo University, 1-1-1 Izumino, Itakura-machi, Ora-gun, Gunma 374-0193, Japan; 5 Atmosphere and Ocean Research Institute, The University of Tokyo, 5-1-5 Kashiwanoha, Kashiwa City, Chiba Prefecture, 277-8564, Japan

**Keywords:** endo-parasites, energy expenditure, flight, accelerometry, seabird, performance-related behaviour

## Abstract

Parasites have profound fitness effects on their hosts, yet these are often sub-lethal, making them difficult to understand and quantify. A principal sub-lethal mechanism that reduces fitness is parasite-induced increase in energetic costs of specific behaviours, potentially resulting in changes to time and energy budgets. However, quantifying the influence of parasites on these costs has not been undertaken in free-living animals. We used accelerometers to estimate energy expenditure on flying, diving and resting, in relation to a natural gradient of endo-parasite loads in a wild population of European shags *Phalacrocorax aristotelis*. We found that flight costs were 10% higher in adult females with higher parasite loads and these individuals spent 44% less time flying than females with lower parasite loads. There was no evidence for an effect of parasite load on daily energy expenditure, suggesting the existence of an energy ceiling, with the increase in cost of flight compensated for by a reduction in flight duration. These behaviour specific costs of parasitism will have knock-on effects on reproductive success, if constraints on foraging behaviour detrimentally affect provisioning of young. The findings emphasize the importance of natural parasite loads in shaping the ecology and life-history of their hosts, which can have significant population level consequences.

## 1. Introduction

Parasites are among the most successful life forms and infect nearly every known animal taxon [1]. It is well known that parasites can have major detrimental fitness consequences on their hosts (e.g. [2–4]). Such consequences may arise because of multiple costs imposed by parasites, such as immune or stress responses or the direct extraction of resources from their hosts [5–7]. These costs are frequently sub-lethal, whereby they alter fitness-related traits, yet these processes are poorly understood because they are challenging to quantify [8]. A principal sub-lethal effect of parasites that can readily be quantified is the impact on performance in terms of movement and foraging. This is a critical mechanism since impairment of these behaviours can have substantial negative fitness consequences [9,10].

Since energy in wild animals is limited [11], there can be a trade-off between allocation of resources to parasite-induced immune responses and performance-related behaviours [12]. Using an energetics framework with energy as the central currency can therefore shape our understanding of how parasites impose behavioural costs on their hosts, and whether hosts compensate for these costs by changing the duration of behaviours to regulate overall energy expenditure. An energetics framework requires the measurement of variation in performance-related behaviours in relation to parasite burdens [13]. However, linking parasitism mechanistically to performance in free-ranging animals has been undertaken in only a very small number of cases and often in tractable invertebrate systems [13].

For most taxa, foraging for resources is tightly linked to fitness [9,14], and can be quantified through its impacts on rates of energy expenditure and gain [15,16]. Foraging is an energetically costly behaviour, especially for breeding individuals commuting between a fixed breeding site and foraging locations, because the energetic costs of travel imposes limits on the allocation of time and resources for self-maintenance and offspring provisioning [17,18]. Even with unlimited energy availability, energy expenditure for foraging and other activities is limited in wild animals [11]. There is evidence that intrinsic constraints may impose an ‘energy ceiling’ in terms of a limit or optimum to daily energy expenditure (DEE, the total energy expenditure of an animal over 24 h) defined as the sum of the cost of activities multiplied by time spent on those activities [19]. As such, to maintain DEE, any increase in costs of a behaviour may be compensated for by reducing the time allocation to that behaviour. Such compensation may then lead to changes in fitness [20,21]. However, these processes have not previously been quantified in free-living animals.

Here, we quantify the costs of nematode endo-parasites on the cost and time allocation of specific behaviours (flying, resting and diving) in breeding European shags *Phalacrocorax aristotelis*. Breeding adult shags are central place foragers commuting between the nest site and foraging grounds, experiencing high energetic costs of foraging activities [22]. Furthermore, recent experimental work has illustrated the critical role of parasitism in European shag reproduction [4,23]. As such, they are a useful study species to test the effects of parasitism on the cost and allocation to key foraging activities. In this study, we aim to quantify the costs of endo-parasites by testing three main hypotheses: (H1) parasite load is linked to an increase in the energetic cost of behaviours; (H2) time allocated to affected behaviours is negatively related to parasite load; and (H3) increases in energetic cost of behaviours will be compensated for by changes in time allocation resulting in no link between parasite load and DEE.

## 2. Material and methods

### (a) Study site and species

The study was carried out on the Isle of May National Nature Reserve, south-east Scotland (56°11′ N, 2°33′ W) during the breeding seasons of 2014–2017. All individuals were part of a long-term population study and are marked with a unique metal ring and a darvic ring for identification. Adults are sexually dimorphic, with males 22% larger than females [24], and are sexed by vocalizations [25]. Populations of European shags *P. aristotelis* are susceptible to nematode gastro-intestinal parasites, in particular anisakid nematodes *Contracaecum rudolphii.* Previous sampling of this population through dissection, faecal egg counts and endoscopy has shown a high prevalence of *C. rudolphii* [4,23,26], though parasite loads vary markedly between individuals [23,26]. Effects are usually sub-lethal, whereby parasites compete with the host for nutrients and initiate costly immune responses [27]. Shags become infected with third stage larvae via their fish diet. Larval worms moult to become sexually mature adults which attach to the lining of the proventriculus and lower oesophagus in the final seabird host [26,27].

### (b) Measuring parasite loads

Adult European shags were captured on the nest using a crook on the end of a long pole. Endoscopy to quantify individual parasite burdens was undertaken in early chick rearing (when the chicks were between 5 and 38 days old). To ensure that individuals had empty stomachs, endoscopy was performed between 03.30 and 07.30, before they had left for their first foraging trip of the day. Worm burdens were counted visually using the endoscope video screen, though counts of worms for burdens of greater than 40 were not possible owing to the number of worms preventing good visibility. Counts of worms greater than this were recorded as greater than 40. These methods were found to be repeatable within an individual across a season [26]. For detailed endoscopy methods see Burthe *et al*. [26]. All endoscopy was performed by trained personnel (S.B.) holding a personal licence operating under a project licence issued by the UK Home Office under the Animals (Scientific Procedures) Act 1986.

### (c) Measuring energy expenditure

All birds were then equipped with tri-axial accelerometers (D3GT, little Leonardo, Tokyo, Japan, AXY3-Depth, Technosmart, Rome, Italy and Gulf Coast Data Concepts X8) to measure the energetic cost of variation in parasite load. Accelerometers ranged in mass from 6.5–9 g but all were less than 0.7% of the minimum shag body weight in this study well within the recommended acceptable limit of logger weights. Accelerometers were set to record at 25 or 50 Hz and attached on the midline of the mid back of individuals (as close to the centre of gravity as possible) using Tesa tape. All birds were successfully recaptured and accelerometers were retrieved after four days of deployment (92 deployments on endoscoped individuals across four years with 57 unique individuals; *n* = 4 in 2014, *n* = 24 in 2015, *n* = 39 in 2016, *n* = 25 in 2017).

Data logger traces were used to differentiate between diving, flying and resting (the three main activities of shags) in two steps. Firstly the Ethographer application, which was developed to classify behaviour states in European shags [28], in IGOR Pro software (Wavemetrics Inc., Portland, OR, USA, 2000, version 6.3.5) was used to assign data as diving (including surface and subsurface periods) or non-diving behaviour, through supervised cluster analysis using *k* means methods on the depth trace [28]. In the case of the accelerometers where depth data were not available, cluster analysis was performed on the surge axis. Secondly, the remaining accelerometer data was assigned as either flight or resting behaviour (either at sea or on land) using frequency histograms of the standard deviation of the heave axis and pitch (calculated over 60 s) [29].

Overall dynamic body acceleration (ODBA) was calculated by first smoothing each of the three acceleration channels with a running mean to represent acceleration primarily owing to gravity. In our study, the running mean was 1 s (i.e. 25 data points for 25 Hz accelerometers) as in [29]. The smoothed value was then subtracted from the corresponding unsmoothed data for that time interval to produce a value of *g* resulting primarily from dynamic acceleration [30]. Derived values were then converted into absolute positive units, and the values from all three axes were summed to give an overall value for dynamic acceleration experienced.

Estimates of oxygen consumption 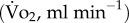 were derived from ODBA values using calibration equations from Hicks *et al*. [31] to determine behavioural specific energy expenditure values for all behavioural bouts. For each individual, we calculated a mean daily rate of oxygen consumption for each behaviour, averaging all bouts of that behaviour on that day. DEE was the sum of the energetic costs of all behavioural bouts within a full 24 h period of activity. Individuals had between one and four of these periods, dependent on the length of the logger deployment. We treated estimates of energy expenditure at the individual level as a measurement, as in previous studies of field energetics using a variety of approaches [32,33].

### (d) Extrinsic variables

We incorporated extrinsic variables that impact on the foraging behaviour of breeding adults since the effect of parasitism in this system varies across environmental conditions [23]. Mean population productivity (measured as the average number of fledged young per incubated nest in a series of long-term monitoring plots from the wider island population) was included as a measure of annual environmental conditions, following [23]. Chick age of the oldest chick in the brood was estimated from wing length at ringing at approximately 20 days of age (a reliable indicator of chick age [34]) and used to back calculate an estimate of age in days at time of logger deployment. Brood size (number of chicks at logger deployment) was also incorporated in analyses, as adult energy expenditure and foraging effort is likely to vary with the total brood mass that must be provisioned [35].

### (e) Statistical analysis

To test H1, we first considered the cost of three behaviours in response to parasite load. We modelled mean daily rate of oxygen consumption for each bird each day 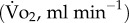 separately for flight, diving and resting behaviours using linear mixed effects models. Parasite load, mean population productivity, brood size and chick age in days were fitted as continuous explanatory variables and we accounted for variation among individuals and years by including individual, year and a year by individual interaction as random factors. Interactions between parasite load and each of the other three explanatory variables were considered. We fitted models for males and females separately owing to non-independence of nest pairs and differing parasite load distributions between sexes and indications that similar parasite loads have different impacts on males and females [4,23]. See the electronic supplementary material, ST1 for a description of model structures and explanations; all possible subsets of fixed effects were considered when running the model selection (subject to the standard restriction that interaction terms are only included alongside the corresponding main effect terms). To test H2 and quantify how the proportion of time spent per day in behaviours changed with parasite load, we modelled logit transformed proportion per day of each behaviour (flight, diving and resting on land) in separate models, using the same set of explanatory variables as in the analysis of costs of behaviours. Finally, to test H3 we modelled DEE using the same model structure as those for the individual behaviours.

In all model sets, model selection was based on Akaike's information criterion (AIC), which penalizes the inclusion of parameters in models, and hence should lead to the removal of unnecessary parameters [36]. The model with the lowest AIC is usually chosen to be the ‘best’ model, but models within two ΔAIC of the lowest value are generally considered to have similar empirical support to that of the best model. All models were fitted using the *lme4* package in R [37,38].

Finally, to investigate the links between energetic costs, behaviours and DEE in full, we used predicted values from our models to estimate the total cost of each behaviour per day in high and low parasite burden scenarios. This was achieved by multiplying the predicted energetic rates by the predicted proportion of time spent in each behaviour under maximum and minimum parasite loads measured in the study. We also summed the total simulated costs of each of the three behaviours to estimate DEE for each parasite burden scenario. This enables us to understand whether changes in costs of behaviours were compensated for via changes in duration of that behaviour.

## 3. Results

### (a) The effect of parasite load on the energetic cost of behaviours

**Figure 1.**
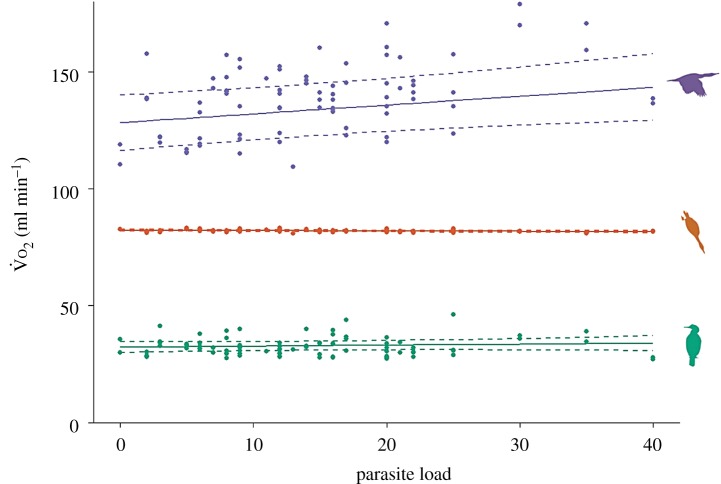
The effect of parasite load on the cost 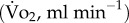 of flight, diving and resting behaviour for female European shags. Solid lines represent predicted values from the best supported model for each behaviour and dashed lines represent 95% confidence intervals. Points represent mean daily rate of oxygen consumption for each behaviour of each bird on each day. Purple is cost of flight, orange is cost of diving and green is cost of resting. All three behaviour predicted lines are extracted from separate behaviour specific models.

In females, an effect of parasite load on the cost of foraging behaviours was detected in all three behaviour specific models; effect sizes varied with the largest effect apparent in flight behaviour (see figure 1 for comparison of all behaviours on the same scale). In males, there was weak evidence for a positive effect of parasitism on flight behaviour but no evidence for the effect of parasitism on dive or rest behaviour.

#### (i) Flight behaviour

The best supported model for the effect of parasitism on the cost of flight in females showed a positive relationship between parasite load, with the cost of flight behaviour increasing by approximately 10% from the minimum to maximum parasite load (see the electronic supplementary material, ST2). This model also incorporated a positive effect of brood age and a negative effect of brood size on the cost of flight (see the electronic supplementary material, figure S2). A positive effect of parasite load was included in all of the six best supported models (within two ΔAIC of the top model). For males, the best supported model included a positive effect of parasite load as well as a positive effect of brood age and an interaction between brood age and parasite load (see the electronic supplementary material, figure S2). However, in this case, parasite load was only included in four of the six best supported models and the null model is 1.53 ΔAIC within the top model, suggesting some caution in interpreting this result (see the electronic supplementary material, ST2).

#### (ii) Dive behaviour

The best supported model for the effect of parasite load on the cost of diving behaviour for females included a negative effect of parasite load, though the effect size was small in that the dive costs at maximum observed parasite loads were just 0.7% lower than at the minimum observed parasite load. Equally some caution must be exercised as parasite load is only included in two of the four best supported models (see the electronic supplementary material, ST3). For males, the best model was the null model.

#### (iii) Rest behaviour

The best supported model for females included a positive effect of parasitism, with the cost of rest increasing by 5% from the minimum to maximum parasite load. This model also incorporated a negative effect of brood size, a positive effect of brood age (see the electronic supplementary material, figure S3) and mean population productivity as well as an interaction between parasitism and mean population productivity (electronic supplementary material, ST4). In lower productivity years there is a greater positive relationship between parasite load and the cost of rest than in high productivity years. However, the null model is only 1.16 ΔAIC within the top model. The best supported model for males was the null model.

### (b) The effect of parasite load on the proportion of time spent in foraging behaviours

**Figure 2.**
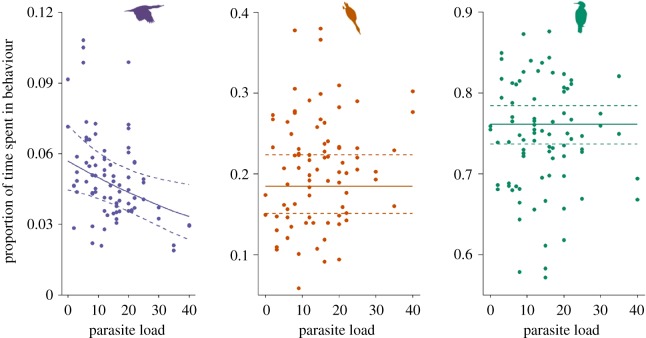
Impact of variation in individual parasite load on the proportion of time spent in flight, diving and rest with respect to other foraging behaviours in female European shags. Solid line represents predicted values from the best supported model for females dashed lines represent 95% confidence intervals. Points represent the proportion of time spent in each behaviour for each bird each day. Different panels represent the three behaviours left to right; flight, diving and rest. Models are fitted separately for each behaviour therefore predicted proportions do not sum to one.

In females, the effect of parasite load was found to be strongly negatively related to the proportion of time spent in flight behaviour but no effect on diving or resting time budgets could be identified (figure 2). In males, there was no evidence for the effect of parasite load on the time budgets of any behaviour.

#### (i) Proportion of time spent in flight

The best supported model showed a negative effect of parasite load on the proportion of time spent in flight for females (electronic supplementary material, ST5) such that individuals with the highest parasite loads spend 44% less time in flight than individuals with the lowest parasite loads (figure 2). The best supported model for males (electronic supplementary material, ST5) did not include parasite load but contained a positive effect of brood age on the proportion of time spent in flight behaviour (see the electronic supplementary material, figure S4).

#### (ii) Proportion of time spent in diving

The best supported model for females included a positive effect of brood age on the proportion of time spent diving (electronic supplementary material, ST6). The best supported model for males also included a positive effect of brood age on the proportion of time spent in diving behaviour (see the electronic supplementary material, figure S5).

#### (iii) Proportion of time spent in resting

The best supported model for females included a negative effect of brood age on the proportion of time spent resting. For males, the best supported model also included a negative effect of brood age on the proportion of time spent resting (electronic supplementary material, ST7).

### (c) The effect of parasite load on daily energy expenditure

There was no evidence for an effect of parasite load on DEE for females or males (electronic supplementary material, ST8). For females, the model containing parasite load was 2.36 AIC units greater than the best-supported model and for males the model containing parasite load was 2.24 AIC units greater than the best-supported model. The best supported models showed only a positive effect of brood age on DEE for both males and females (see the electronic supplementary material, figure S7).

### (d) Predicted behavioural costs

**Figure 3.**
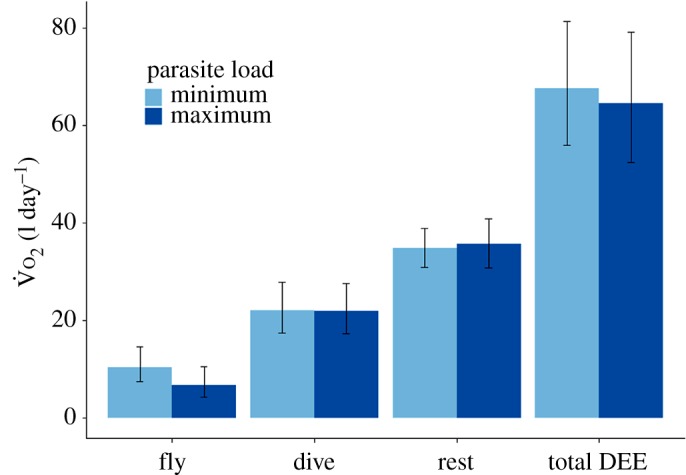
Predicted total energy expenditure 
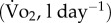
 for female shags in each behaviour per day with 95% confidence intervals. Total energy expenditure values for each behaviour are calculated based on predicted values for the proportion of time spent in each behaviour multiplied by the predicted rate of energy expenditure for that behaviour. Total DEE is calculated by summing the total energy expenditure for the three behaviours. Light blue bars represent predictions based on a minimum natural parasite load scenario and dark blue bars represent predictions based on maximum parasite loads. If behavioural costs and time budgets balance out there should be no difference in total behavioural energy expenditure between maximum and minimum parasite load scenarios.

Despite costs of flight increasing with parasite load, the maximum parasite load scenario has a lower total energy expenditure spent in flight. Given that the proportion of flight per day decreases with parasite load, this suggests that individuals with high parasite loads reduce flight time more than is actually required (figure 3).

## 4. Discussion

In this study, we quantified the energetic cost of parasitism to individuals of a free-living population, to our knowledge for the first time. We detected a change in the energetic cost of behaviours and time budgets owing to variation in natural endo-parasite load in female European shags but no relationship between parasite load and DEE in either sex. Specifically, in support of H1 and H2, we found the cost of flight to be higher in females with higher parasite loads and these individuals spent a smaller proportion of their time in this behaviour than females with lower parasite loads. We found a small effect of parasitism on the cost of diving and resting behaviour in female shags but no change to their time energy budgets as a result of parasite load. Overall, in support of H3, this compensation suggests that European shags are limited in their DEE and must compensate for increased activity costs by reducing their duration; this suggests energetic trade-offs exist between performance-related behaviours and immune responses to parasitism.

Flight usually comprises a large proportion of an individual's daily energy budget in volant birds with flapping flight [39]. For species such as the European shag, where energy expenditure is limited by a tight energy budget or optimal energy ceiling, we would expect a reduction in investment in more costly behaviours. Accordingly, we found that individuals with higher parasite burdens and greater flight costs spent a smaller proportion of time in flight than individuals with low parasite burdens. Similarly, experimentally parasitized honeybees were found to perform a lower number of daily flights than control individuals [40].

While flight behaviour in any species requires large energy outputs and efficient muscle use, endo-parasites can affect nutrient assimilation and muscle efficiency essential for flight [41]. Shags like other cormorant species are continuous flapping flyers and have limited flight performance as a trade-off to their diving ability [42]. Consequently, any damage to muscle efficiency or feather quality could have significant impacts on flight costs. There is evidence of the negative impacts of endo-parasites on feather repair and development as well as stress on feather quality in other species of birds [43,44]. Additionally preen gland size is negatively related to immune function which, via preen oil production, can also impact feather condition function [43,44].

By measuring the costs of active behaviours such as flight and diving behaviour, we can gain an understanding of how these high cost behaviours might link to energetic limits. However, resting behaviour costs should partially reflect the maintenance costs of an individual and be elevated with an increase in immune response [45,46]. That we see a small increase in the cost of resting behaviour with increased parasite load could be an indication of increased maintenance cost. However it is known that accelerometry is not a good proxy for resting costs in active animals [47] and further measurements of resting or basal metabolic rate using a more appropriate approach is recommended.

In establishing the response of DEE to parasite load our results suggest that adult shags may have an optimum energy ceiling, as demonstrated in several other seabird species during the breeding season [20,48]. This optimum energy ceiling means that any increase in cost to an activity must be counteracted by either a reduction in duration of that activity or a reduction in cost of other activities. This assertion is supported by our finding that individuals with more costly flight behaviour spent a lower proportion of their time per day in flight, ensuring that DEE was unaffected. In calculating total behavioural costs (figure 3) we found individuals with high parasite loads decreased the proportion of time spent in flight more than would be predicted solely from the increase in the cost of this behaviour, as there was an overall decrease in the mean total cost of flight per day (figure 3). This trade-off between flight costs and allocation suggests that changes in the proportion of time spent in flight may also be compensating for other costs or changes in energy use. We would expect that any increase in maintenance costs arising from an immune response, that were not measureable in this study, would require additional compensation through reduced activity [20]. As flight is the most costly behaviour that shags can use to divest energy expenditure, it may be that maintenance costs in the highly parasitized individuals are being compensated for with the extra reduction in flight time.

Previous work has shown that extrinsic variables such as wind, presence of food in the stomach and other environmental conditions can affect foraging energetics, behaviour and breeding success [49,50]. Our findings are consistent with this finding, providing additional evidence that environmental drivers are important in energy use. Our finding that brood age was positively related to flight costs in females is expected, since adults provisioning large chicks return from foraging grounds with larger food loads, which incurs higher costs on the inbound flight [50]. This relates to the experimental evidence of Reed *et al*. [4] where females spent more time foraging with increasing age of their offspring when they were relieved of their parasite load. Similarly, DEE also increased with brood age which we interpret to mean that shags have an optimal energy ceiling at any given stage of the breeding season, perhaps reflecting the demands of provisioning the chicks and/or investment made to that point. DEE of provisioning birds often increases to accommodate extra energetic needs of offspring [51], however the scope to which DEE can be raised further in response to parasites appears to be limited, either owing to physiological, extrinsic or other factors [15].

The negative relationship found between brood size and cost of flight behaviour may relate to the quality of the individuals, such that individuals with larger broods are likely to be higher quality individuals in better condition with more energy to assign to maintenance such as feather condition and therefore may experience lower costs in flight. It is known that parasite effects can vary with environmental conditions [23]; therefore it is important to note that effects in this study were all from data collected in years with high mean population productivity (our proxy for environmental conditions). We would expect to see more extreme effects of parasitism in lower productivity years where poor foraging conditions can cause individuals to be under more severe energetic constraint.

We consistently found limited evidence for the effect of parasite load on energy budgets or costs of behaviours in male European shags. This difference among sexes corroborates previously established differences in investment in reproduction and consequently different constraints on energy expenditure in females compared to males across many species [52]. Experimental work in shags also shows stronger effects on foraging time in females than males when parasites are removed [4]. The larger of the sexes often show higher foraging efficiency in bird species [49]. Consistent with the assumption that females (the smaller sex in shags) are more constrained energetically, only females showed an energetic and behavioural response to parasitism despite both sexes showing a positive increase in DEE with brood age. These sex differences in the impact of parasites could have consequences for survival; indeed, previous work has shown that during winter female shags have lower survival than males [53].

## 5. Conclusion

In most parasite-host systems there is marked heterogeneity with respect to parasite load within the population, which often leads to demographic differences among individuals [54]. In this study, we demonstrated that, parasite load was related to energy expenditure and time budgets of foraging behaviours, which may be a key process underpinning the demographic consequences of parasites. This work demonstrates that energetics is a powerful framework to aid the understanding of individual-level mechanisms driving life-history. This study provides a potential mechanism behind experimental evidence of sex biased fitness effects of parasitism in a free-ranging population. The findings emphasize the importance of natural parasite loads in shaping the ecology and life-history of their hosts, which can have significant population level consequences [8].

## Supplementary Material

Supplementary Figures and Tables

## Data accessibility

The data and script for this study are archived in the repository figshare and were made available to editors and reviewers upon submission (https://doi.org/10.6084/m9.figshare.6231560).
