# Supplementary material for: The energetic cost of parasitism in a wild population
Source: Proc Biol Sci. 2018 May 30;285(1879):20180489. doi: 10.1098/rspb.2018.0489 (PMC5998108; doi:10.1098/rspb.2018.0489)
Supplement: Supplementary Figures and Tables [file rspb20180489supp1.docx]

Online Electronic Supplementary Information for:

**The energetic cost of parasitism in a wild population**

Olivia Hicks^1, *^, Sarah J. Burthe^2^, Francis Daunt^2^_,_ Mark Newell^2^, Adam Butler^3^, Motohiro Ito^4^, Katsufumi Sato^5,^ Jonathan A. Green^1^

^1^ School of Environmental Sciences, University of Liverpool

^2^ Centre for Ecology & Hydrology

^3^ Biomathematics and Statistics Scotland

^4^Department of Applied Biosciences, Faculty of Life Sciences, Tokyo University

^5^Atmosphere and Ocean Research Institute, The University of Tokyo

**
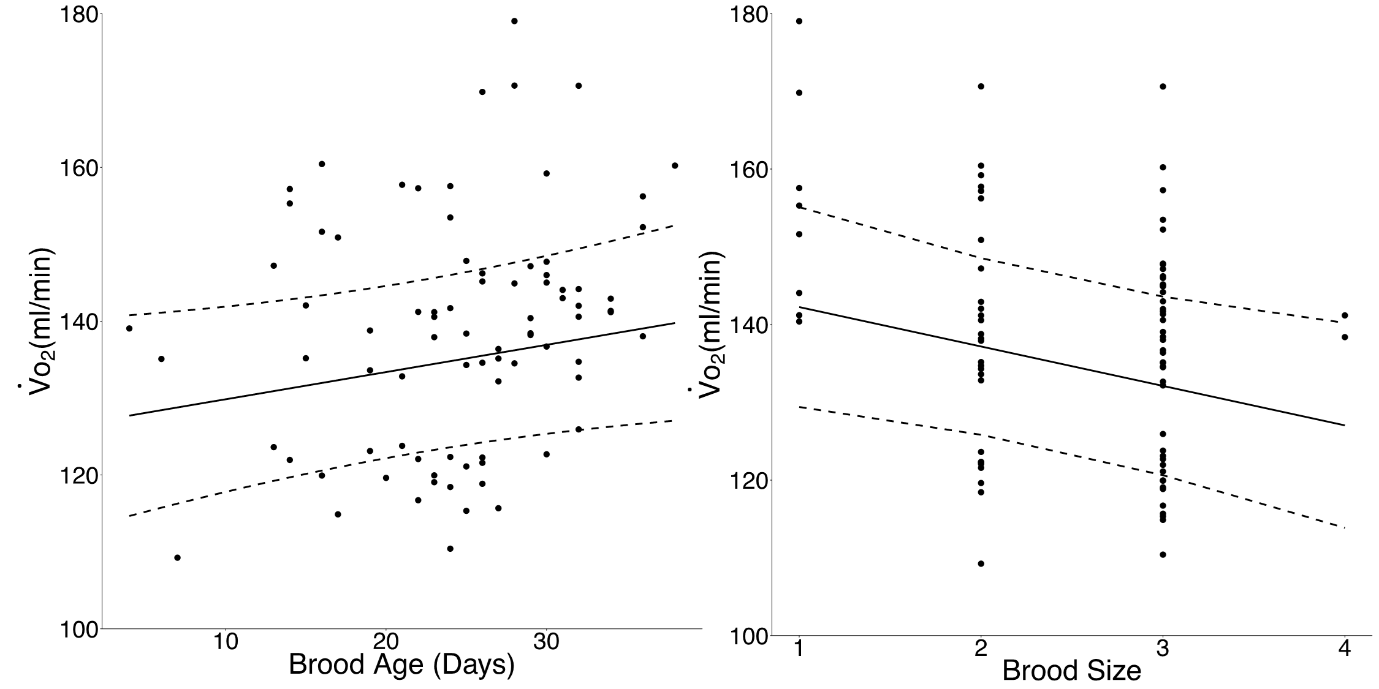
**

**Fig. S1. Impact of variation in brood size and brood age (days) on the energetic cost (V.o_2_ ml/min) of flight in females.** Solid lines represent predicted values from the best supported model and dashed lines represent 95% confidence intervals. Other variables in the best supported model were fixed at their mean to look solely at the effect of the parameter of interest.


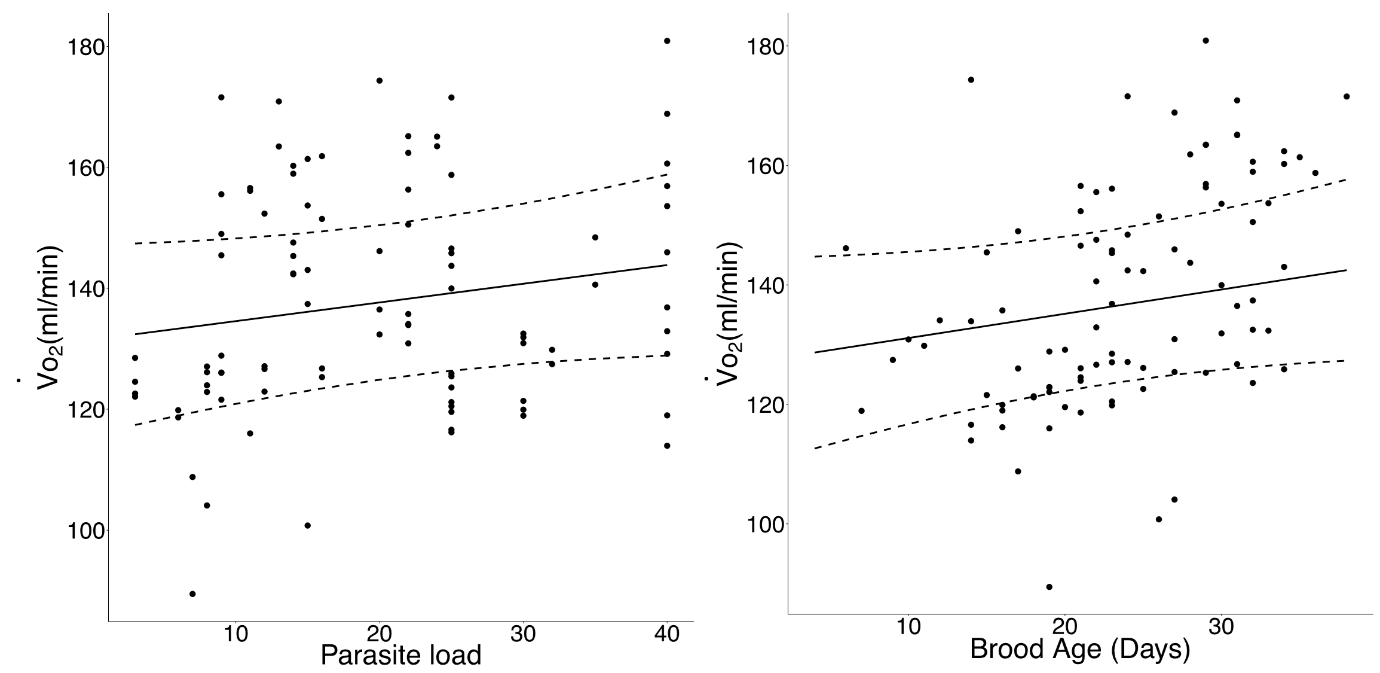


**Fig. S2. Impact of variation in parasite load and brood age on the energetic cost (V.o_2_ml/min) of flight in males.** Solid lines represent predicted values from the best-supported model and dashed lines represent 95% confidence intervals. Other variables in the best supported model were fixed at their mean to look solely at the effect of the parameter of interest.

**
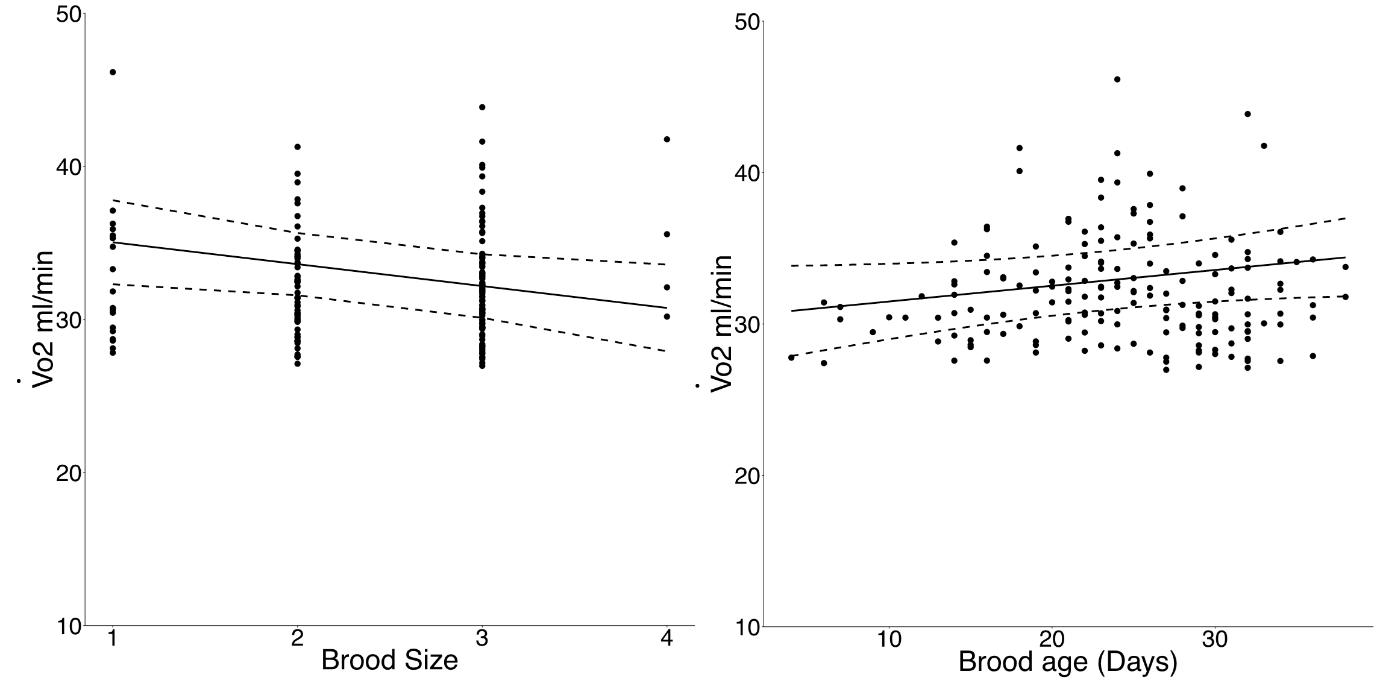
**

**Fig. S3. Impact of brood size and brood age on the energetic cost (**V**.o_2_ ml/min) of rest in females.** Solid lines represent predicted values from the best-supported model and dashed lines represent 95% confidence intervals.

**
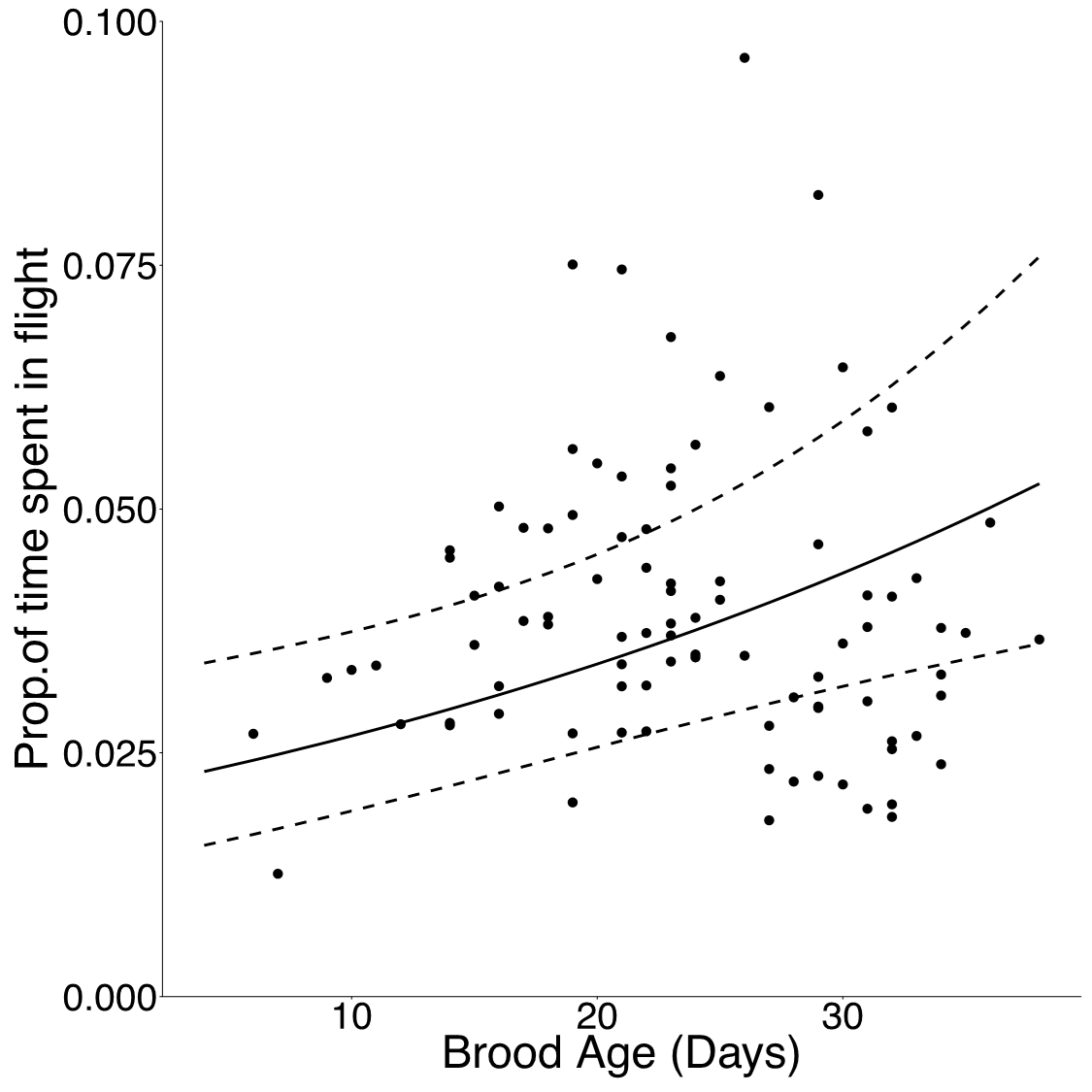
**

**Fig. S4. The effect of brood age (days) on the proportion of time spent flying in male shags.** Solid lines represent predicted values for the best supported model and dashed lines represent 95% confidence intervals.

**
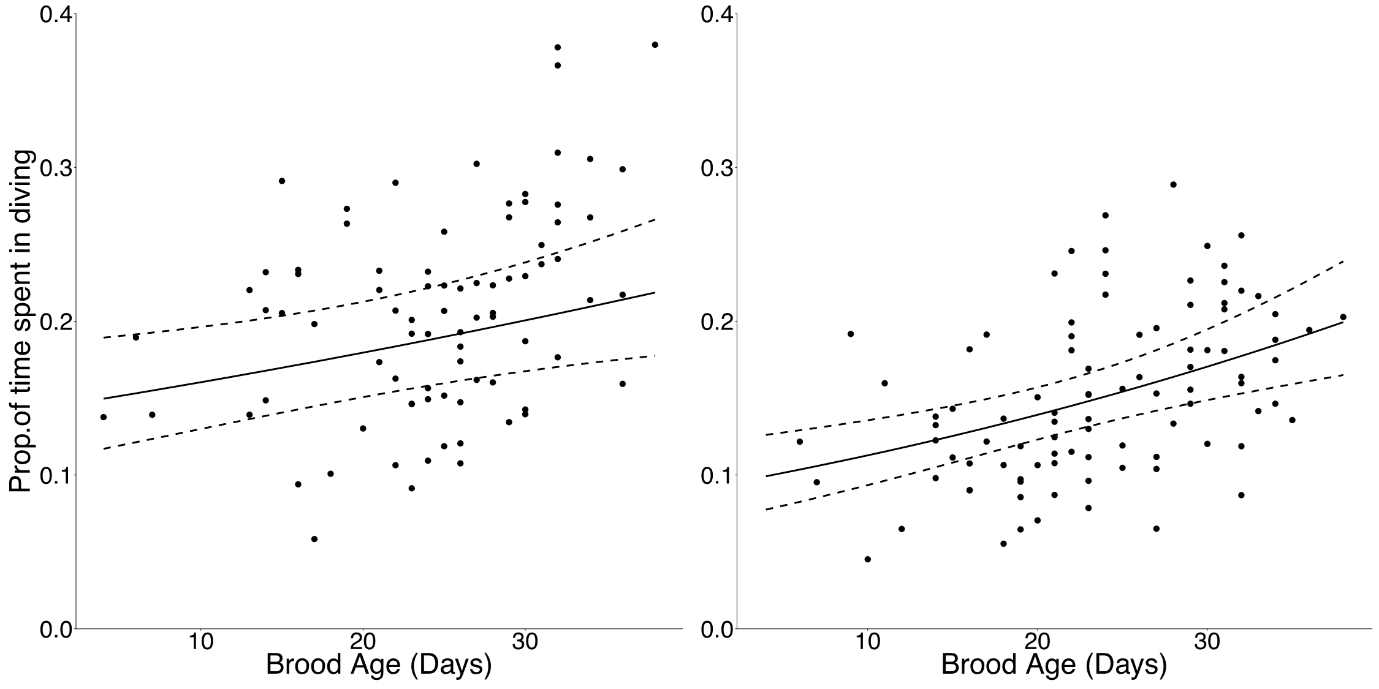
**

**Fig. S5. The effect of brood age (days) on the proportion of time spent diving in female and male shags (left to right).** Solid lines represent predicted values for the best supported model and dashed lines represent 95% confidence intervals.

**
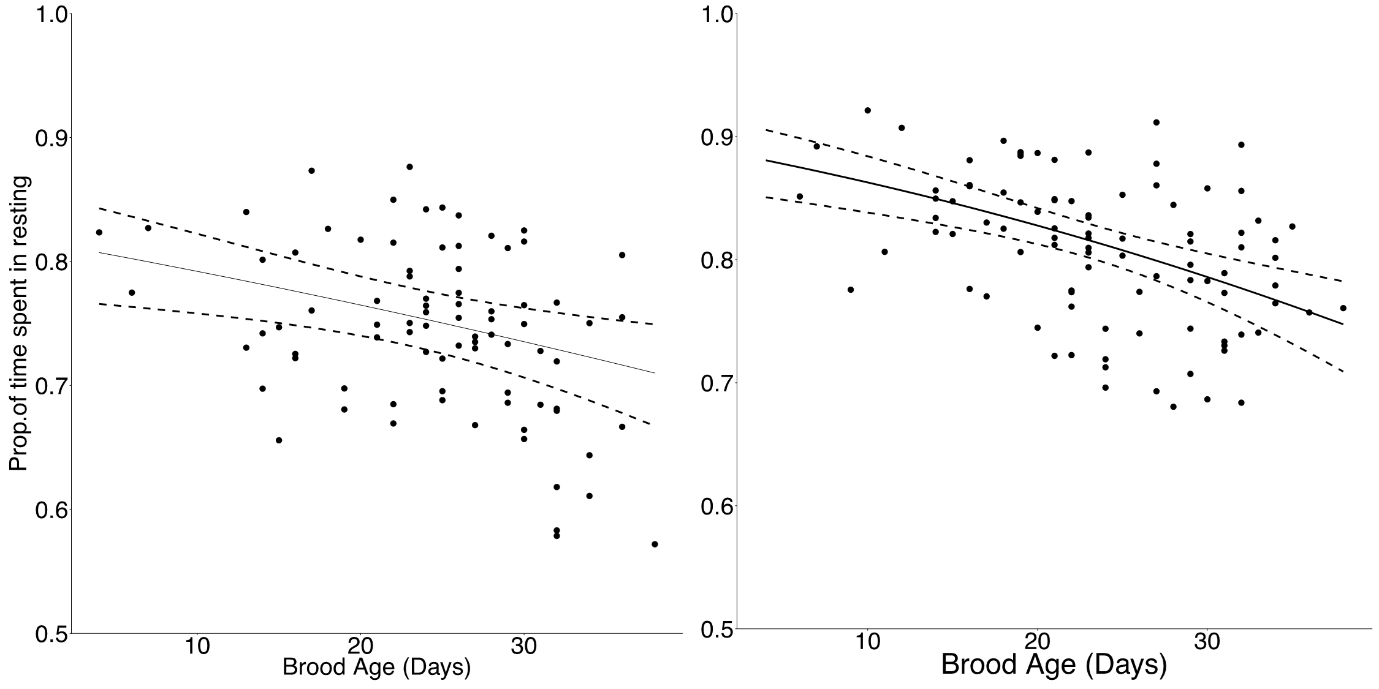
**

**Fig. S6. The effect of brood age (days) on the proportion of time spent resting in female and male shags (left to right).** Solid lines represent predicted values for the best supported model and dashed lines represent 95% confidence intervals.

**
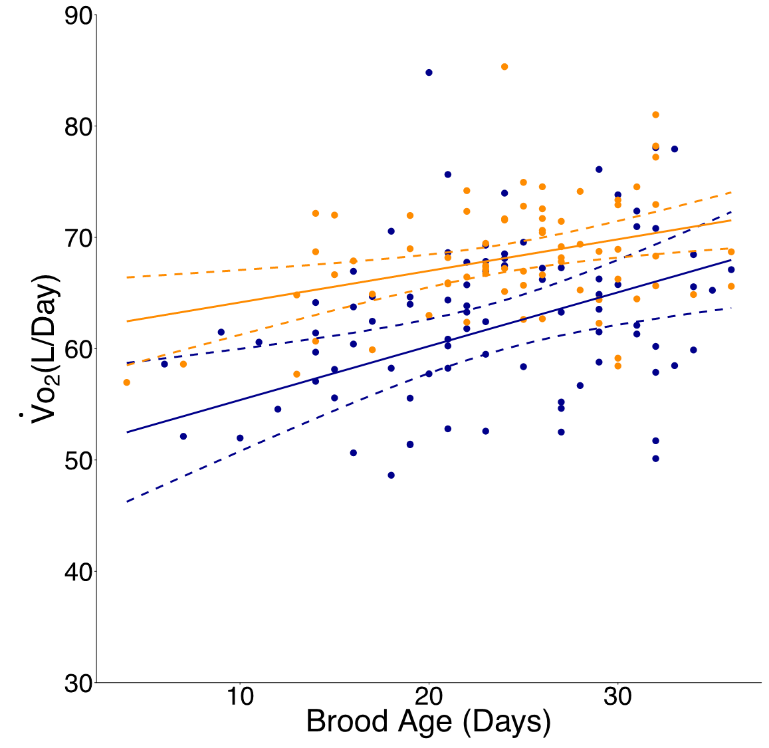
**

**Fig. S7. The effect of brood age on the Daily energy expenditure in adult shags.** Solid lines represent predicted values for the best supported model and dashed lines represent 95% confidence intervals males in dark blue and females in orange.

**ST 1. Model variables and structure for the three questions we ask in this study. Models were run separately for males and females on all occasions.** Random effects are included in all models; the set of explanatory variables to include is selected by fitting all possible subsets of these variables, and comparing the resulting model fits using AIC.

| Type |  | Variable |
| --- | --- | --- |
| Response variables | Qu. 1: Does parasite load drive an increase in the energetic cost of foraging behaviours | Energetic cost of flight  Energetic cost of diving  Energetic cost of resting |
|  |  |  |
|  |  |  |
|  | Qu. 2: Does parasite load drive changes in time budgets, specifically a reduction in the proportion of time spent in foraging behaviours | Proportion of time spent flying  Proportion of time spent diving  Proportion of time spent resting |
|  |  |  |
|  |  |  |
|  | Qu. 3: Does Daily Energy Expenditure vary in relation to parasite load, or are any changes in costs of behaviours compensated for by a change in time budget of that behaviour. | Daily Energy Expenditure |
| Explanatory variables |  | Parasite load |
|  |  | Brood Size |
|  |  | Mean pop. Prod |
|  |  | Brood Age |
|  | Effects of parasites could vary with reproductive demand of the brood, it is also important to account for brood size as it is known to influence foraging behaviour | Parasite load * Brood Size |
|  | Effects of parasites have been found to vary with prevailing environmental conditions | Parasite load * Mean pop. Prod |
|  | Effects of parasites could vary with reproductive demand of the brood, it is also important to account for brood age as it is known to influence foraging behaviour. | Parasite load * Brood Age |
| Random structure |  | Individual |
|  |  | Year |
|  |  | Individual * Year |

**ST2. The top ten best-supported models for the effect of parasite load on the cost of flying behaviour for male and female European shags**

|  | **(Int)** | **Parasite load** | **Brood Size** | **Mean pop. Prod** | **Brood Age** | **Parasite load * Brood Size** | **Parasite load * Brood Age** | **df** | | **logLik** | **AICc** | **ΔAIC** | **weight** |
| --- | --- | --- | --- | --- | --- | --- | --- | --- | --- | --- | --- | --- | --- |
|  |  |  |  |  |  |  |  |  |  |  |  |  |  |
| **Female** | -0.20 | 0.23 | -0.22 |  | 0.15 |  |  | 8 | -66.83 | | 151.60 | 0.00 | 0.20 |
|  | -0.29 | 0.22 | -0.17 |  |  |  |  | 7 | -68.39 | | 152.30 | 0.66 | 0.14 |
|  | -0.30 | 0.21 | -0.17 |  |  | -0.14 |  | 8 | -67.36 | | 152.70 | 1.05 | 0.12 |
|  | -0.22 | 0.23 | -0.21 |  | 0.12 | -0.10 |  | 9 | -66.28 | | 153.00 | 1.41 | 0.10 |
|  | -0.26 | 0.25 |  |  |  |  |  | 6 | -70.01 | | 153.10 | 1.52 | 0.09 |
|  | -0.24 | 0.20 | -0.20 |  | 0.16 |  | 0.12 | 9 | -66.38 | | 153.20 | 1.62 | 0.09 |
|  | -0.14 | 0.23 | -0.23 | 0.20 | 0.14 |  |  | 9 | -66.64 | | 153.70 | 2.14 | 0.07 |
|  | -0.29 | 0.18 | -0.19 |  | 0.13 | -0.14 | 0.17 | 10 | -65.42 | | 153.90 | 2.29 | 0.06 |
|  | -0.29 |  | -0.23 |  | 0.15 |  |  | 7 | -69.25 | | 154.00 | 2.39 | 0.06 |
|  | -0.19 | 0.26 |  |  | 0.10 |  |  | 7 | -69.26 | | 154.00 | 2.42 | 0.06 |
| **Male** | -0.08 | 0.19 |  |  | 0.17 |  | 0.23 | 8 | -86.42 | | 190.50 | 0.00 | 0.23 |
|  | -0.09 |  |  |  | 0.21 |  |  | 6 | -89.25 | | 191.50 | 0.93 | 0.15 |
|  | -0.18 | 0.18 |  |  | 0.20 |  |  | 7 | -88.35 | | 192.00 | 1.46 | 0.11 |
|  | -0.16 |  |  |  |  |  |  | 5 | -90.69 | | 192.10 | 1.53 | 0.11 |
|  | -0.25 | 0.19 |  |  |  |  |  | 6 | -89.63 | | 192.20 | 1.69 | 0.10 |
|  | -0.16 | 0.18 |  | -0.27 | 0.19 |  | 0.26 | 9 | -86.13 | | 192.40 | 1.87 | 0.09 |
|  | -0.08 | 0.19 | -0.01 |  | 0.17 |  | 0.23 | 9 | -86.41 | | 193.00 | 2.44 | 0.07 |
|  | -0.15 | 0.15 |  | -0.40 | 0.21 | 0.18 | 0.22 | 10 | -85.43 | | 193.50 | 2.98 | 0.05 |
|  | -0.13 |  |  | -0.11 | 0.21 |  |  | 7 | -89.21 | | 193.70 | 3.19 | 0.05 |
|  | -0.09 |  | -0.03 |  | 0.22 |  |  | 7 | -89.22 | | 193.70 | 3.21 | 0.05 |

**ST3. The top ten best-supported models for the effect of parasite load on the cost of diving behaviour for male and female European shags**

|  | **(Int)** | **Parasite load** | **Brood Size** | **Mean pop. Prod** | **Brood Age** | **Parasite load * Mean. Pop** | **df** | **logLik** | **AICc** | **ΔAIC** | **weight** |
| --- | --- | --- | --- | --- | --- | --- | --- | --- | --- | --- | --- |
|  |  |  |  |  |  |  |  |  |  |  |  |
| **Female** | 0.15 | -0.27 |  |  |  |  | 6 | -106.96 | 227.00 | 0.00 | 0.24 |
|  | 0.22 |  |  |  |  |  | 5 | -108.44 | 227.70 | 0.64 | 0.18 |
|  | 0.21 | -0.27 |  | 0.31 |  |  | 7 | -106.39 | 228.30 | 1.26 | 0.13 |
|  | 0.30 |  |  | 0.34 |  |  | 6 | -107.95 | 229.00 | 1.98 | 0.09 |
|  | 0.14 | -0.28 | -0.06 |  |  |  | 7 | -106.88 | 229.20 | 2.23 | 0.08 |
|  | 0.16 | -0.27 |  |  | 0.03 |  | 7 | -106.93 | 229.40 | 2.33 | 0.08 |
|  | 0.23 |  |  |  | 0.05 |  | 6 | -108.37 | 229.90 | 2.83 | 0.06 |
|  | 0.23 | -0.27 |  | 0.27 |  | -0.12 | 8 | -105.99 | 229.90 | 2.90 | 0.06 |
|  | 0.17 |  | -0.10 |  |  |  | 6 | -108.52 | 230.10 | 3.13 | 0.05 |
|  | 0.22 | -0.29 | -0.10 | 0.36 |  |  | 8 | -106.16 | 230.30 | 3.25 | 0.05 |
| **Male** | -0.39 |  |  |  |  |  | 5 | -95.19 | 201.10 | 0.00 | 0.21 |
|  | -0.34 |  |  |  | 0.17 |  | 6 | -94.22 | 201.40 | 0.35 | 0.18 |
|  | -0.46 | 0.15 |  |  |  |  | 6 | -94.59 | 202.20 | 1.10 | 0.12 |
|  | -0.30 |  |  | 0.22 |  |  | 6 | -94.75 | 202.50 | 1.41 | 0.10 |
|  | -0.41 | 0.15 |  |  | 0.16 |  | 7 | -93.67 | 202.60 | 1.57 | 0.10 |
|  | -0.28 |  |  | 0.16 | 0.15 |  | 7 | -93.98 | 203.30 | 2.21 | 0.07 |
|  | -0.39 |  | -0.01 |  |  |  | 6 | -95.19 | 203.30 | 2.28 | 0.07 |
|  | -0.34 |  | -0.06 |  | 0.19 |  | 7 | -94.11 | 203.50 | 2.46 | 0.06 |
|  | -0.36 | 0.15 |  | 0.24 |  |  | 7 | -94.21 | 203.70 | 2.65 | 0.06 |
|  | -0.46 | 0.15 | 0.00 |  |  |  | 7 | -94.59 | 204.50 | 3.43 | 0.04 |

**ST4. The top ten best-supported models for the effect of parasite load on the cost of resting behaviour for male and female European shags**

|  | **(Int)** | **Parasite load** | **Brood Size** | **Mean pop. Prod** | **Brood Age** | **Parasite load * Brood Size** | **Parasite load * Mean pop.** | **df** | **logLik** | **AICc** | **ΔAIC** | **weight** |
| --- | --- | --- | --- | --- | --- | --- | --- | --- | --- | --- | --- | --- |
|  |  |  |  |  |  |  |  |  |  |  |  |  |
| **Female** | 0.19 | 0.12 | -0.28 | 0.17 | 0.19 |  | -0.37 | 10 | -112.00 | 247.10 | 0.00 | 0.14 |
|  | 0.04 | 0.05 | -0.16 |  |  | -0.38 |  | 8 | -114.67 | 247.30 | 0.23 | 0.13 |
|  | 0.16 | 0.10 | -0.22 | 0.16 |  |  | -0.40 | 9 | -113.44 | 247.30 | 0.29 | 0.12 |
|  | 0.16 | 0.12 |  | 0.05 |  |  | -0.38 | 8 | -114.73 | 247.40 | 0.35 | 0.12 |
|  | 0.13 | 0.08 | -0.21 | 0.15 |  | -0.22 | -0.30 | 10 | -112.36 | 247.80 | 0.73 | 0.10 |
|  | 0.07 | 0.08 | -0.22 |  | 0.18 | -0.33 |  | 9 | -113.70 | 247.90 | 0.81 | 0.10 |
|  | 0.07 |  |  |  |  |  |  | 5 | -118.72 | 248.20 | 1.16 | 0.08 |
|  | 0.16 | 0.10 | -0.26 | 0.16 | 0.17 | -0.18 | -0.30 | 11 | -111.31 | 248.30 | 1.28 | 0.08 |
|  | 0.09 |  | -0.26 |  | 0.24 |  |  | 7 | -116.46 | 248.40 | 1.36 | 0.07 |
|  | 0.18 | 0.14 |  | 0.04 | 0.14 |  | -0.36 | 9 | -114.00 | 248.50 | 1.40 | 0.07 |
| **Male** | -0.08 |  |  |  |  |  |  | 5 | -125.70 | 262.10 | 0.00 | 0.26 |
|  | -0.06 |  |  |  | 0.16 |  |  | 6 | -125.01 | 263.00 | 0.90 | 0.17 |
|  | -0.07 |  | 0.10 |  |  |  |  | 6 | -125.25 | 263.50 | 1.38 | 0.13 |
|  | -0.06 | -0.08 |  |  |  |  |  | 6 | -125.47 | 263.90 | 1.83 | 0.10 |
|  | -0.07 |  |  | 0.04 |  |  |  | 6 | -125.66 | 264.30 | 2.21 | 0.09 |
|  | -0.06 |  | 0.06 |  | 0.13 |  |  | 7 | -124.86 | 265.00 | 2.94 | 0.06 |
|  | -0.05 | -0.05 |  |  | 0.15 |  |  | 7 | -124.91 | 265.10 | 3.03 | 0.06 |
|  | -0.06 |  |  | 0.00 | 0.16 |  |  | 7 | -125.01 | 265.30 | 3.24 | 0.05 |
|  | -0.05 | -0.07 | 0.10 |  |  |  |  | 7 | -125.04 | 265.40 | 3.30 | 0.05 |
|  | -0.07 |  | 0.10 | 0.01 |  |  |  | 7 | -125.25 | 265.80 | 3.71 | 0.04 |

**ST5. The top ten best-supported models for the effect of parasite load on the proportion of time spent in flight per day for male and female European shags**

|  | **(Int)** | **Parasite load** | **Brood Size** | **Mean pop. Prod** | **Brood Age** | **Parasite load * Brood Size** | **Parasite load * Mean pop. Prod** | **df** | **logLik** | **AICc** | **ΔAIC** | **weight** |
| --- | --- | --- | --- | --- | --- | --- | --- | --- | --- | --- | --- | --- |
|  |  |  |  |  |  |  |  |  |  |  |  |  |
| **Female** | 0.18 | -0.35 |  |  |  |  |  | 6 | -89.78 | 192.70 | 0.00 | 0.27 |
|  | 0.20 | -0.32 | 0.16 |  |  |  |  | 7 | -89.03 | 193.60 | 0.89 | 0.17 |
|  | 0.22 | -0.35 |  | 0.19 |  |  |  | 7 | -89.41 | 194.30 | 1.63 | 0.12 |
|  | 0.19 | -0.34 |  |  | 0.06 |  |  | 7 | -89.67 | 194.80 | 2.16 | 0.09 |
|  | 0.29 |  |  |  |  |  |  | 5 | -92.42 | 195.60 | 2.94 | 0.06 |
|  | 0.22 | -0.33 | 0.14 | 0.13 |  |  |  | 8 | -88.87 | 195.70 | 3.02 | 0.06 |
|  | 0.30 |  | 0.21 |  |  |  |  | 6 | -91.29 | 195.70 | 3.03 | 0.06 |
|  | 0.21 | -0.36 |  | 0.23 |  |  | 0.13 | 8 | -88.89 | 195.70 | 3.07 | 0.06 |
|  | 0.19 | -0.32 | 0.17 |  |  | -0.05 |  | 8 | -88.97 | 195.90 | 3.23 | 0.05 |
|  | 0.20 | -0.32 | 0.16 |  | 0.02 |  |  | 8 | -89.02 | 196.00 | 3.32 | 0.05 |
| **Male** | -0.27 |  |  |  | 0.43 |  |  | 6 | -122.62 | 258.20 | 0.00 | 0.31 |
|  | -0.27 |  | 0.14 |  | 0.37 |  |  | 7 | -122.19 | 259.70 | 1.47 | 0.15 |
|  | -0.24 | -0.10 |  |  | 0.42 |  |  | 7 | -122.42 | 260.10 | 1.93 | 0.12 |
|  | -0.30 | -0.13 |  |  | 0.52 | -0.27 |  | 8 | -121.23 | 260.10 | 1.94 | 0.12 |
|  | -0.24 |  |  | 0.09 | 0.42 |  |  | 7 | -122.59 | 260.50 | 2.26 | 0.10 |
|  | -0.24 | -0.07 | 0.13 |  | 0.37 |  |  | 8 | -122.09 | 261.90 | 3.66 | 0.05 |
|  | -0.24 |  | 0.14 | 0.07 | 0.37 |  |  | 8 | -122.16 | 262.00 | 3.81 | 0.05 |
|  | -0.22 | -0.13 |  | 0.25 | 0.51 | -0.30 |  | 9 | -120.98 | 262.10 | 3.89 | 0.04 |
|  | -0.30 | -0.11 | 0.10 |  | 0.48 | -0.25 |  | 9 | -121.05 | 262.20 | 4.04 | 0.04 |
|  | -0.21 | -0.10 |  | 0.08 | 0.41 |  |  | 8 | -122.39 | 262.50 | 4.25 | 0.04 |

**ST6. The top ten best-supported models for the effect of parasite load on the proportion of time spent in diving behaviour per day for male and female European shags**

|  | **(Int)** | **Parasite load** | **Brood Size** | **Mean pop. Prod** | **Brood Age** | **Parasite load * Mean prod.** | **Parasite load * Brood Size** | **df** | **logLik** | **AICc** | **ΔAIC** | **weight** |
| --- | --- | --- | --- | --- | --- | --- | --- | --- | --- | --- | --- | --- |
|  |  |  |  |  |  |  |  |  |  |  |  |  |
| **Female** | 0.35 |  |  |  | 0.23 |  |  | 6 | -93.06 | 199.20 | 0.00 | 0.32 |
|  | 0.33 |  |  | -0.13 | 0.24 |  |  | 7 | -92.91 | 201.30 | 2.08 | 0.11 |
|  | 0.40 | 0.07 |  |  | 0.21 |  | -0.25 | 8 | -91.69 | 201.30 | 2.10 | 0.11 |
|  | 0.35 |  | 0.02 |  | 0.22 |  |  | 7 | -93.04 | 201.60 | 2.34 | 0.10 |
|  | 0.35 | 0.01 |  |  | 0.23 |  |  | 7 | -93.06 | 201.60 | 2.39 | 0.10 |
|  | 0.36 | 0.08 |  | -0.15 | 0.23 | 0.19 | -0.29 | 10 | -89.31 | 201.70 | 2.44 | 0.09 |
|  | 0.31 | 0.00 |  | -0.10 | 0.25 | 0.18 |  | 9 | -91.19 | 202.80 | 3.62 | 0.05 |
|  | 0.37 | 0.08 |  | -0.18 | 0.22 |  | -0.26 | 9 | -91.38 | 203.20 | 4.00 | 0.04 |
|  | 0.30 |  |  |  |  |  |  | 5 | -96.24 | 203.30 | 4.04 | 0.04 |
|  | 0.32 |  | 0.03 | -0.15 | 0.23 |  |  | 8 | -92.86 | 203.70 | 4.44 | 0.03 |
| **Male** | -0.27 |  |  |  | 0.41 |  |  | 6 | -107.05 | 227.10 | 0.00 | 0.20 |
|  | -0.33 |  |  | -0.20 | 0.46 |  |  | 7 | -105.99 | 227.30 | 0.21 | 0.18 |
|  | -0.26 |  | 0.13 |  | 0.34 |  |  | 7 | -106.36 | 228.00 | 0.95 | 0.12 |
|  | -0.33 |  | 0.14 | -0.24 | 0.40 |  |  | 8 | -105.21 | 228.10 | 1.05 | 0.12 |
|  | -0.21 | -0.12 |  |  | 0.39 |  |  | 7 | -106.49 | 228.30 | 1.20 | 0.11 |
|  | -0.29 | -0.11 |  | -0.23 | 0.43 |  |  | 8 | -105.61 | 228.90 | 1.85 | 0.08 |
|  | -0.20 | -0.12 | 0.13 |  | 0.33 |  |  | 8 | -105.70 | 229.10 | 2.03 | 0.07 |
|  | -0.29 | -0.11 | 0.14 | -0.26 | 0.37 |  |  | 9 | -104.74 | 229.60 | 2.55 | 0.06 |
|  | -0.23 | -0.12 |  |  | 0.42 |  | -0.05 | 8 | -106.43 | 230.60 | 3.48 | 0.04 |
|  | -0.19 | -0.12 | 0.09 |  | 0.35 | 0.11 |  | 9 | -105.23 | 230.60 | 3.54 | 0.03 |

**ST7. The top ten best-supported models for the effect of parasite load on the proportion of time spent in resting behaviour per day for male and female European shags**

|  | **(Int)** | **Parasite load** | **Brood Size** | **Mean pop. Prod** | **Brood Age** | **Parasite load * Mean pop. Prod** | **Parasite load * Brood Age** | **df** | **logLik** | **AICc** | **ΔAIC** | **weight** |
| --- | --- | --- | --- | --- | --- | --- | --- | --- | --- | --- | --- | --- |
|  |  |  |  |  |  |  |  |  |  |  |  |  |
| **Females** | -0.44 |  |  |  | -0.26 |  |  | 6 | -92.31 | 197.70 | 0.00 | 0.22 |
|  | -0.48 | -0.09 |  | 0.10 | -0.27 | -0.23 | 0.33 | 10 | -87.43 | 197.90 | 0.18 | 0.20 |
|  | -0.45 |  |  | 0.12 | -0.28 |  |  | 7 | -91.81 | 199.10 | 1.39 | 0.11 |
|  | -0.47 | -0.03 |  |  | -0.23 |  | 0.28 | 8 | -90.73 | 199.40 | 1.68 | 0.09 |
|  | -0.44 | -0.01 |  | 0.06 | -0.31 | -0.23 |  | 9 | -89.54 | 199.60 | 1.82 | 0.09 |
|  | -0.44 |  | -0.07 |  | -0.24 |  |  | 7 | -92.15 | 199.80 | 2.06 | 0.08 |
|  | -0.43 | 0.04 |  |  | -0.26 |  |  | 7 | -92.27 | 200.00 | 2.31 | 0.07 |
|  | -0.50 | -0.10 |  | 0.16 | -0.26 |  | 0.32 | 9 | -89.88 | 200.20 | 2.50 | 0.06 |
|  | -0.48 | -0.09 | -0.03 | 0.11 | -0.27 | -0.22 | 0.32 | 11 | -87.40 | 200.50 | 2.79 | 0.05 |
|  | -0.44 |  | -0.09 | 0.16 | -0.26 |  |  | 8 | -91.54 | 201.00 | 3.29 | 0.04 |
| **Males** | 0.37 |  |  |  | -0.45 |  |  | 6 | -110.50 | 234.00 | 0.00 | 0.20 |
|  | 0.38 |  | -0.19 | 0.20 | -0.43 |  |  | 8 | -108.24 | 234.20 | 0.22 | 0.18 |
|  | 0.38 |  |  | 0.15 | -0.48 |  |  | 7 | -109.59 | 234.50 | 0.53 | 0.15 |
|  | 0.37 |  | -0.13 |  | -0.41 |  |  | 7 | -109.85 | 235.00 | 1.04 | 0.12 |
|  | 0.34 | 0.11 |  |  | -0.45 |  |  | 7 | -110.01 | 235.30 | 1.36 | 0.10 |
|  | 0.35 | 0.08 | -0.18 | 0.19 | -0.43 |  |  | 9 | -107.93 | 236.00 | 2.04 | 0.07 |
|  | 0.35 | 0.09 |  | 0.14 | -0.48 |  |  | 8 | -109.23 | 236.10 | 2.19 | 0.07 |
|  | 0.34 | 0.10 | -0.13 |  | -0.41 |  |  | 8 | -109.36 | 236.40 | 2.46 | 0.06 |
|  | 0.33 | 0.12 |  |  | -0.50 |  | 0.09 | 8 | -109.76 | 237.20 | 3.26 | 0.04 |
|  | 0.34 | 0.10 | -0.08 |  | -0.41 | -0.15 |  | 9 | -108.68 | 237.50 | 3.54 | 0.03 |

**ST8. The top ten best-supported models for the effect of parasite load on Daily energy expenditure for male and female European shags**

|  | **(Int)** | **Parasite load** | **Brood Size** | **Mean pop. Prod** | **Brood Age** | **Parasite load * Brood Age** | **df** | **logLik** | **AICc** | **ΔAIC** | **weight** |
| --- | --- | --- | --- | --- | --- | --- | --- | --- | --- | --- | --- |
|  |  |  |  |  |  |  |  |  |  |  |  |
| **Female** | 0.34 |  |  |  | 0.29 |  | 6 | -91.46 | 196.00 | 0.00 | 0.31 |
|  | 0.34 |  | -0.13 |  | 0.33 |  | 7 | -90.73 | 196.90 | 0.93 | 0.19 |
|  | 0.34 |  |  | -0.02 | 0.29 |  | 7 | -91.41 | 198.30 | 2.30 | 0.10 |
|  | 0.34 | 0.02 |  |  | 0.29 |  | 7 | -91.45 | 198.40 | 2.36 | 0.09 |
|  | 0.37 | 0.06 | -0.16 |  | 0.30 | -0.27 | 9 | -89.25 | 198.90 | 2.93 | 0.07 |
|  | 0.36 | 0.06 |  |  | 0.26 | -0.22 | 8 | -90.51 | 198.90 | 2.94 | 0.07 |
|  | 0.34 |  | -0.15 | 0.04 | 0.33 |  | 8 | -90.66 | 199.20 | 3.24 | 0.06 |
|  | 0.34 | 0.00 | -0.13 |  | 0.33 |  | 8 | -90.73 | 199.40 | 3.37 | 0.06 |
|  | 0.34 | 0.02 |  | -0.03 | 0.29 |  | 8 | -91.40 | 200.70 | 4.72 | 0.03 |
|  | 0.36 | 0.07 |  | -0.05 | 0.26 | -0.23 | 9 | -90.34 | 201.10 | 5.10 | 0.02 |
| **Male** | -0.27 |  |  |  | 0.40 |  | 6 | -122.82 | 258.60 | 0.00 | 0.29 |
|  | -0.26 |  | 0.13 |  | 0.35 |  | 7 | -122.21 | 259.70 | 1.11 | 0.17 |
|  | -0.27 |  |  | -0.09 | 0.42 |  | 7 | -122.51 | 260.30 | 1.69 | 0.13 |
|  | -0.26 |  | 0.18 | -0.14 | 0.37 |  | 8 | -121.49 | 260.60 | 2.05 | 0.10 |
|  | -0.28 | 0.04 |  |  | 0.39 |  | 7 | -122.78 | 260.80 | 2.24 | 0.10 |
|  | -0.26 | 0.01 |  |  | 0.48 | -0.16 | 8 | -122.09 | 261.80 | 3.25 | 0.06 |
|  | -0.27 | 0.05 | 0.14 |  | 0.35 |  | 8 | -122.13 | 261.90 | 3.33 | 0.06 |
|  | -0.28 | 0.05 |  | -0.10 | 0.41 |  | 8 | -122.42 | 262.50 | 3.91 | 0.04 |
|  | -0.28 | 0.06 | 0.18 | -0.15 | 0.36 |  | 9 | -121.33 | 262.80 | 4.17 | 0.04 |
|  | -0.26 | 0.02 | 0.12 |  | 0.43 | -0.14 | 9 | -121.58 | 263.20 | 4.66 | 0.03 |
